# Using Generative Artificial Intelligence (AI) to Compare Commission on Osteopathic College Accreditation (COCA) and Liaison Committee on Medical Education (LCME) Standards

**DOI:** 10.7759/cureus.100411

**Published:** 2025-12-30

**Authors:** Jason S Hedrick, Scott A Cottrell, Linda S Nield, Norman Ferrari

**Affiliations:** 1 Department of Medical Education, West Virginia University School of Medicine, Morgantown, USA

**Keywords:** accreditation, ai, ai chatbots, chatgpt, coca, google gemini, grok 3, large language models (llm), lcme, medical education

## Abstract

Background

The Liaison Committee on Medical Education (LCME) for medical doctors (MDs) and Commission on Osteopathic College Accreditation (COCA) for osteopathic doctors (DOs) serve as the accrediting bodies for US medical schools. Although conventional wisdom suggests a number of differences between the two sets of standards, there are relatively few studies that parse out substantive distinctions in greater detail.

Objective

The objective of this research project was to identify significant differences between the LCME and COCA standards and elements.

Design

This study utilized three generative chatbots, ChatGPT-4o (Open AI, California, US), Gemini 2.0 Flash (Google DeepMind, London, England and Google Research, California, US), and Grok 3 (xAI, California, US), to ascertain key distinctions. An identical prompt was given to each chatbot. The chatbots ranked the key differences between the standards from most significant difference to least significant difference.

Key results

Twenty-three themes were identified. The chatbots collectively agreed on two distinct differences: osteopathic manipulative medicine in DOs requirements, and differing approaches to diversity, equity, and inclusion between the standards. From there, results varied, but included, for example, publishing student outcomes at DO schools, leadership requirements, research requirements, and student narrative expectations, to name a few.

Conclusions

Discourse has posited that the standards have grown necessarily similar. However, within the elements’ details, evident differences remain. Although the chatbot results drew clear distinctions, some responses were less compelling as significant, including, for example, student access to mental healthcare, interaction with residents, and the reporting of major changes to the accreditor. There were several limitations, including the chatbots selected, which were among popular and publicly available systems. Two errors were produced by and reconciled with the chatbots. The study only included the standards and elements, and not additional requirements such required narrative prompts associated with elements. Therefore, the clear intent of each element may not have been recognized by the chatbots.

## Introduction

Within the United States (US), there are two degree options to become a practicing physician: a Doctor of Medicine (MD) degree, accredited through the Liaison Committee on Medical Education (LCME), or a Doctor of Osteopathic Medicine (DO) degree, accredited through the Commission on Osteopathic College Accreditation (COCA). The purpose of this explorative study is to investigate the extent to which DO and MD degree accreditation standards may be disparate. Accordingly, the objective is to utilize generative AI chatbots to identify and characterize the most significant differences between LCME and COCA accreditation standards and elements, and to determine whether these tools reveal areas of divergence not consistently highlighted in existing literature. Recognizing the distinctions between the DO and MD programs could have implications for efforts to coalesce the two degree types under one accreditation system.

Both types could also benefit from this analysis, as potential gaps in accreditation standards could be linked to vulnerabilities in the learning environment and educational outcomes. Moreover, identifying commonalities and distinctions makes it possible to explore programmatic differences and, in turn, potential variations in the graduates’ abilities to demonstrate knowledge and skills specific to residency programs, which require various levels of diagnostic-oriented care and procedural skills. However, using generative AI to help ascertain gaps or differences requires educators to approach results with caution and prudence. 

Literature

The US MD degree accreditation can be traced back to at least 1844, when the Medical Society of the State of New York raised a few concerns, including variability in program duration, rigor, and licensing [1]. Several false starts into standardization eventually received momentum with the establishment of the American Medical Association (AMA) and later the Association of American Medical Colleges (AAMC), along with growing state medical board uniformity [1]. It was not until 1942 that the AMA and AAMC officially formed the LCME as the accrediting authority for MD degree programs [1]. In comparison, osteopathic medicine originated as an alternative to contemporary medical practices of the 1870s [2]. Dr. Andrew Taylor Still offered an alternative medical philosophy in which he argued the current focus of medicine sought to treat disease processes, but instead, it should treat the entire body [2]. Dr. Still founded the first osteopathic college later that century and the creation of the American Association of College of Osteopathic Medicine (AACOM) followed [2]. Several iterations of oversight culminated in the recognition of the American Osteopathic Association (AOA) as the DO accreditation agency [3]. Ultimately, COCA was established in 2004 as the DO accrediting body, but still under the guise of AOA [3]. Training in osteopathic manipulative medicine or therapy (OMM or OMT) is a core distinction of training for DO graduates compared to MDs. OMM includes 40 techniques that can be used to “manipulate” the body for health outcomes [4].

In 2023, of the 1.01 million medical school graduates in the US, 11.5% held a DO, while 88.5% were MDs [5]. Medical school enrollment is on the rise, particularly in the DO realm. Between the academic years 2012 and 13 and 2022 and 23, overall medical student enrollment grew by nearly 29%. MD student enrollment grew by 18%, and DO student enrollment grew by 69% [5]. Growth in both degree tracks is attributed to class size increases at established medical schools and the formation of new accredited schools. COCA now declares that one in four medical students in the US attends one of 42 osteopathic medical schools [6]. The other three out of four students attend one of the 160 LCME schools with full, preliminary, or provisional accreditations status [7]. In 2023, the pool of US physician graduates was composed of about 27% DOs and 73% MDs [8].

Once graduates earn an MD or DO degree, most continue their education and complete a residency program. All specialties have limited residency positions, and some are most restrictive (e.g., orthopedic surgery, plastic surgery) which results is a competitive application process. There is variability in how many DO and MD graduates are accepted across residency specialties. No major specialty in the US is made up of more than 25% of DO graduates [5]. Although DOs practice across the medical specialty spectrum, the majority (57%) practice in specialties designated as primary care [9]. The largest share of DO practice falls into sports medicine (a subspecialty of family medicine), where 22.7% of practicing physicians are DO graduates [5]. The family medicine specialty is composed of 21.9% DOs, and physical medicine and rehabilitation (PM&R) is 19.0% DO graduates [5]. No other specialty nears 20% of the DOs in the workforce, with pain medicine/management (13.7%), hospice and palliative medicine (13.5%), and emergency medicine (13%) having the fourth, fifth, and sixth largest share of DO physicians [5]. Many specialties have fewer than 5% DOs working in the field, many of which are surgical specialties. The smallest share of DOs includes thoracic surgery (3.1%), urology (3.0%), ophthalmology (2.6%), neurosurgery (2.5%), plastic surgery (2.0%), and radiation oncology (1.8%) [5].

Since inception, the LCME considered a potential role in the accreditation process or oversight of osteopathic schools, but never gained the consensus to do so [1]. A few commentaries have debated key differences between the accreditation standards of both entities [10-13]. Initial discussion revolved around using accreditation standards to assess quality among graduates [10-12]. Such a question is exceedingly complex and would necessarily need to entail an assessment of graduate and provider outcomes long after medical school [11,12]. There is limited published data in all age groups and in all specialties comparing outcomes of MD versus DO-treated patients. Two studies, one regarding surgeons and the other hospitalists, reported no differences in 30-day mortality rates, readmissions, and length of stays in patients aged 65 years and older who underwent common surgical procedures or were hospitalized for varied medical conditions [14,15]. Jenkins et al. used pass rates of the American Board of Internal Medicine (ABIM)-certifying examination as a measure of residency program quality. They reported that programs which were dominated by MD residents had significantly higher pass rates by 4.0 percentage points, while the programs dominated by DO residents programs had significantly lower pass rates [16]. Regarding philosophy of care, Johnson and Kurtz described the “blurred lines” between allopathic and osteopathic physicians based on survey results completed by 955 DO respondents [17]. The majority of the respondents did not perceive any distinguishing philosophies or behaviors between their allopathic counterparts or themselves [17]. While findings from an older study revealed that osteopathic physicians were more likely to describe themselves as “socioemotionally oriented” rather than “technoscientifically oriented” compared to their allopathic counterparts, both groups of physicians expressed equally negative attitudes toward the competence of primary care physicians [18]. Moreover, there is evidence that OMT is underutilized or rarely used for treatment by DO graduates, a key distinction of the osteopathic educational model [19].

Both MD and DO program leadership have demonstrated signs of cooperation. As late as 2015, the LCME and COCA have agreed to collaborate on common issues, including shared clinical learning spaces [1]. In 2014, the American Osteopathic Association of Colleges of Osteopathic Medicine (AOACOM) began a merger with the Accreditation Council for Graduate Medical Education (ACGME) for residency program accreditation partially resulting from osteopathic school and graduate growth outpacing the number of positions available in DO residency programs [20]. Ahmed et al. proffer that much has changed since Wood & Hahn examined key differences between DO and MD accreditation standards, including the merger of graduate medical education (GME), and the intent of the standards now share many commonalities making a single accreditation system feasible [10,13].

This study contributes to extant literature that has explored key differences between the structures of the accrediting bodies and whether standards and their more specific elements are unique to or intersect between DO and MD programs. As for structure, differences pointed out more than a decade ago (e.g., COCA lacking student representatives) have now been remedied and oversight boards are relatively similar [10,13]. The literature has presented unique differences in the standards. Some differences noted in 2009 include qualifications for leadership, clearer expectations for the academic environment in LCME standards, the role of residents in medical student education, and the expectation for DO graduates to have passed the Comprehensive Osteopathic Medical Licensing Examination (COMLEX-USA) Level I and II by the time of graduation [10]. Others added, among additional differences, greater research opportunities at LCME institutions, differing approaches to diversity, equity, and inclusion (DEI) within both sets of standards, and LCME heightened expectations of clinical learning site quality with clearly defined patient types [11].

Ahmed et al. acknowledge a number of these key differences but recognize a concordant drift, in many ways, to parity as opposed to disparity [13]. Notably, expectations for medical students to have resident exposure has been augmented in the COCA standards [13]. Research expectations and requirements at osteopathic colleges has also increased (including the necessitation of OMT related research) [13]. COCA has also moved more in line with LCME related to DEI, going beyond requirements for antidiscrimination to expectations for inclusivity [13]. Of course, the omnipresent difference is COCA’s requirement for OMT training (and training with osteopaths) as well as expectations for primary care doctors in leadership [13]. However, the authors further extrapolate that, “major areas (of divergence) would include the issue of licensing exams and OMM staff and faculty requirements. With the growing debate on single licensing exams and the exponential decrease in osteopathic physicians utilizing OMM, it does not appear these areas represent major barriers to implementing single accreditation at the undergraduate medical education level” [13].

Untangling the differences between accreditation requirements of both COCA and LCME are complicated, even though both entities accredit schools that graduate practicing physicians who are both eligible for the same GME training, can prescribe the same medications, and have the same scope of practice. Some have explored the differences between the two training pathways, including the differing accreditation standards. However, to our knowledge, no publication has presented a comprehensive list of differences and similarities of standards [12]. In recent years, the accrediting bodies have seemingly become more similar than divergent. For nearly two decades, the literature has debated and explored key differences; however, we seek to leverage a new tool, generative artificial intelligence (AI) chatbots. We ask, what are significant differences between LCME and COCA standards and elements as determined by a sample of publicly available AI systems?

This research does not necessarily attempt to answer fraught questions of quality or assess COCA and LCME merger feasibility. Our research aims to utilize a new tool to gauge key differences. Generative chatbots allows for the opportunity to reduce our own biases in an analysis of the standards. Others have drawn comparisons between COCA and LCME standards, and beginning with those similarities and differences may seem like a logical starting point. However, by using these innovative tools, we can seek new areas of divergence that the current literature may not have explored. 

## Materials and methods

We utilized a comparative analysis with three generative AI chatbots to compare standards and elements. Due to potential variability of responses, we chose three common and publicly available systems. We selected the free versions of ChatGPT-4o (OpenAI, California, US), Gemini 2.0 Flash (Google DeepMind, London, England and Google Research, California, US), and Grok 3 (xAI, California, US), due to their similar usability and accessibility [21-23]. These three systems rely on large language models (LLMs) and have relatively recent version updates. We downloaded the 2025-26 Functions and Structure of a Medical School (published August 2024 for survey in the 2025-2026 academic year, effective July 1, 2025), which contain the LCME standards and elements as well as the Accreditation of Colleges of Osteopathic Medicine: COM Continuing Accreditation Standards (Effective September 26, 2023 (Revised October 1, 2024)) from their respective websites [24,25]. The project leader subsequently purged all extraneous information (e.g., front matter, table of contents, glossary) from the document, leaving only the standards and elements. Initially, we piloted a robust query with ChatGPT-4o that asked to assess similarities, differences, and an analysis of how each set of standards could be strengthened. This query was exploratory and used to assess the general feasibility of the project. We then narrowed the prompt to specifically look at differences between the sets of standards. In March 2025, we submitted the documents and the following prompt to the three AI systems.

Main LLM prompt

The prompt stated: List the 10 most significant differences between the LCME and COCA standards and elements, with one being the most significant and 10 being the least significant. Specifically reference the standards and elements where possible.

Responses from the three chatbots were reviewed determining if the standards and elements were referenced correctly and had overall general accuracy and plausibility of differences. Each difference was mapped and grouped by theme. Some errors were noted in the responses. These included, for example, incorrectly referencing elements or claiming a requirement was or was not a part of a set of standards. As a result, we followed up with the chatbots when errors were perceived, and corrections were made by the chatbots. Relevant responses from this query are included in the appendix, including follow-up queries related to perceived errors and necessary clarification. Otherwise, we did not complete multiple runs of this specific prompt.

## Results

The 30 unique responses of identified differences (10 per each chatbot) were grouped by theme based on content and intent on the response (Table 1), totaling 23 themes.

**Table 1:** Most significant differences between the accreditation standards identified by the chatbots (including the ranking) The data has been represented as N. Greatest identified differences by each of the chatbots are ranked from one through 10, with one being the area of greatest difference and 10 being the least area of difference among the 10 listed. COMLEX-USA: Comprehensive Osteopathic Medical Licensing Examination of the United States; DEI: Diversity, Equity, and Inclusion; GME: Graduate Medical Education; OMM: Osteopathic Manipulative Medicine; OPP: Osteopathic Principles and Practices.

Themes	Chat GPT-4o	Google Gemini	Grok 3
Academic freedom		10	
Affiliation agreements	2		
Clinical education requirements		7	
COMLEX-USA			2
Complaint resolution		6	
Cultural competence	5		
Dean’s qualifications		2	
DEI office and approaches	9	5	3
Faculty credentials		8	
Faculty pay equity reviews	7		
Financial audits	4		
GME support			9
GME transparency	6		
Mandatory reporting of substantial changes	8		
Mental health services			10
Narrative assessment	3		8
OMM core competencies and principles	1	1	1
OMM research		4	4
Primary care and OMM/OPP leadership qualifications		3	7
Program duration			5
Resident participation in education			6
Student debt outcomes		9	
Study space, lounge, call rooms	10		

Across the 30 responses, there were only two instances in which all three chatbots listed a common difference. In two instances, two chatbots shared a similar difference. Other than these, ChatGPT-4o and Gemini shared no other similar responses, ChatGPT-4o and Grok 3 shared one other similar response, and Gemini and Grok 3 shared two other similar responses. In 18 instances, the responses were unique.

We noted two errors in total, one each with ChatGPT-4o and Grok 3. The errors were corrected in Table 1 as they were reconciled with the chatbot. ChatGPT-4o initially noted that COCA does not require affiliation agreements. When queried about this assertation, the system altered its response to indicate key differences between COCA and LCME affiliation agreement requirements. Grok 3 initially listed COCA 7.7 as including a requirement regarding formative and summative assessment. This error was remedied by the chatbot, noting reference should be made to COCA 7 and 7.1.

The differences between COCA and LCME standards/elements are further delineated in Table 2. 

**Table 2:** Comparison of COCA and LCME standards by identified themes The left column includes themes identified by the three AI chatbots. The columns "COCA Standard/Language" and "LCME Standard/Language" detail the specific standard or element being reference for this listed difference as well as a brief explanation. ABMS: American Board of Medical Specialties; AOA: American Osteopathic Association; COCA: Commission on Osteopathic College Accreditation COM: Colleges of Osteopathic Medicine; COMLEX-USA: Comprehensive Osteopathic Medical Licensing Examination of the United States; DCI: Data Collection Instrument; DEI: Diversity Equity and Inclusion; DO: Doctor of Osteopathy; GME: Graduate Medical Education; LCME: Liaison Committee on Medical Education; OMM: Osteopathic Manipulative Medicine; OPP: Osteopathic Principles and Practice; USMLE: United States Medical Licensing Examination.

Topic/Difference	COCA standard/language	LCME standard/language
Academic freedom	12.8 references academic freedom but does not provide a definition.	LCME does not have a comparable standard or element.
Affiliation agreements	COCA 6.9 notes that COM’s must, “Provide executed affiliation agreements that support the clinical educational experience for its students."	LCME 1.4 states, “Written agreements are necessary with clinical affiliates that are used regularly for required clinical experiences.” The LCME element is specific with five additional requirements.
Clinical education requirements	COCA is prescriptive in defining expectations for clinical education. The standards define that the core must include family medicine, internal medicine, general surgery, and pediatrics (and soon, obstetrics & gynecology and psychiatry). Faculty must be, or eligible to be, board-certified (AOA or ABMS) in the given specialty.	LCME 6.3 is the most comparable element but does not require this level of specificity.
COMLEX	COCA requires students to pass COMLEX-USA I & II.	LCME does not explicitly require the passing the USMLE Step 1 or 2 for graduation. LCME 8.4 notes that schools provide evidence of meeting national norms of accomplishment, which includes pass rate metrics for both USMLE Step 1 & 2 (qualified in the standards’ glossary). The glossary was not included in the analysis.
Complaint resolution	COCA 2.4 specifies the schools must have a policy and system to collect confidential accreditation related complaints	No relevant standard/element.
Cultural competence	COCA 6.12 is parsimonious noting, “A COM must incorporate diversity, equity, and inclusion into its curriculum to the extent permitted by law."	LCME 7.6 is a robust element with required instruction on bias and five specific content areas.
Dean’s qualifications	COCA 2.1 is specific in requirements (e.g., earned DO degree, length of experience).	LCME 2.2 is vague, listing abstract qualifications (e.g., “qualified by education, training, and experience”).
DEI office and approaches	COCA 5.2 includes a robust diversity standard. Further, element 5.5 requires a DEI office at each campus. COCA 7.9 notes, “A COM must offer DEI training to employed faculty and staff at least annually to the extent permitted by law." COCA frequently requires DEI, “to the extent permitted by law.”	LCME 3.3 requires “policies and practices… to achieve mission-appropriate diversity outcomes among students” and to foster “diversity among qualified applicants." LCME does not have an expectation of a required DEI office or annual training.
Faculty credentials	COCA 7.1 indicates that all faculty physicians at non-core rotation sites that are not or have not been AOA or ABMS board certified (or not eligible to be), must have their credentials reviewed and approved by the college.	LCME 4 and elements require schools to have mechanisms in place to ensure faculty are qualified to deliver the curriculum, among other expectations. The LCME standard does not offer board certification expectations.
Faculty pay equity reviews	COCA 7.8 includes requirements to, “review pay and rank parity every three years consistent with its mission-appropriate diversity outcomes among its faculty."	LCME does not have a comparable requirement.
Financial audits	COCA 3.4 states that a required “annual independent audit” is required of all schools.	LCME 5.1 does not state this requirement explicitly, although financial audits are a part of the standard LCME review process and data collection instrument (DCI) submission.
GME support	COCA 10.2 and 10.3 require colleges to “provide a mechanism to assist new and existing GME programs in meeting requirements for accreditation” and “A COM must provide a mechanism to assist GME programs in meeting requirements of osteopathic recognition." COCA 10.1 notes that, “A COM must have policies, procedures, personnel, and budgetary resources to support the continuum of osteopathic education, including graduate medical education and continuing medical education."	The LCME makes no comparable requirements of GME support.
GME transparency	COCA 11.4b notes, “A COM must continually publish publicly the placement rates of its students in (GME) programs."	The LCME does not have a related element.
Mandatory reporting of substantial changes	COCA 1.1 notes a requirement for substantive change related to school mission, and 3.1 requires notice if there is a change in status related to Title IV programs. Otherwise, this requirement is formally missing from the elements, but the COCA website does list several “substantive change policies and procedures” and a relevant form for various modifications (e.g., branch campus addition, change in hour calculations, class size). COCA does not have a comparable element to LCME 5.12, but there is some expectation for major change reporting.	LCME 5.12 requires schools notify LCME of major changes, which include enrollment adjustments (class size), resource decline, curriculum modifications, the addition of campuses, and changes with clinical affiliations.
Mental health services	COCA 9.8 states that, “A mental health care provider must be accessible 24 hours a day, 365 days a year, from all locations where the students receive education from the COM.”	LCME 12.3 does not mandate this expectation for around-the-clock services, despite having a requirement for access to services.
Narrative assessment	COCA 11 and 11.1 indicate that schools must collect assessment data for students to ensure competency but is not explicit in the data required. COCA 7 notes a requirement for formative and summative assessment, which does not provide specificity in that expectation.	LCME 9.5 requires “narrative descriptions of student performance in required courses/clerkships when interaction permit.”
OMM core competencies and principles	OMM principles and training is embedded in various requirements, including curriculum, research, and leadership.	No relevant standard or element.
OMM research	COCA 8.3 states, “A COM must demonstrate how its research and/or scholarly activity includes and/or incorporates OMM and OPP.”	Element 3.2, community of scholars and LCME element 7.3 research instruction requirement are not specific to a type of research in the way that COCA 8.3 requires OMM and OPP.
Primary care and OMM/OPP leadership qualifications	COCA 7.4 details that clinical leaders to be board certified osteopathic physicians and 7.5 mandates specific credentials for full-time OMM/OPP leaders.	There is not a comparable LCME requirement that details this specificity in leadership positions, particularly because the COCA elements relate to OMM training.
Program duration	COCA 6.3 requires that school policies denote that students may not exceed “150% of the standard time to achieve the degree (six years following matriculation) and describe any exceptions to the 150%-time limit.”	The LCME (6.8), requires programs span at least 130 weeks of instruction. COCA 6.3 is referencing policies for standard time to student completion of the DO degree.
Resident participation in education	COCA 6.10 notes experiences must include resident interaction in “at least one rotation” which establishes a minimum, possibly to align with the DO model of ambulatory or community hospital settings. COCA 6.10 falls in a list of three requirements that must be satisfied, presenting it with other training needs. COCA 6.10 falls within the curriculum standard, which instead indicates a curricular requirement as opposed to an education environment factor.	LCME 3.1 notes that resident physicians must be present in “one or more required clinical experiences” it highlights that an integrated experience is expected that may be common in teaching hospitals or large medical centers. LCME 3.1 falls within the academic and learning environments standard
Student debt outcomes	COCA 11.3 requires “publish(ed) data on the debt load and student loan default rates of its institutions.”	LCME 12.1 does not include a comparable expectation.
Study space, lounge, call rooms	COCA 4 and 4.1 simply mandate adequate learning resources with list specificity.	LCME 12.1 does not include a comparable expectation.

## Discussion

The chatbots presented 23 themes that explored a broad swath of COCA and LCME standards. In only two instances did all three chatbots share a common theme. Most notably, the mention of OMM training and principles was the most significant key difference between the sets of standards. This finding is expected and promotes the validity of the results. It would be expected that the AI systems would designate this difference, and the systems did so by noting it as the most significant. OMM/OMT-related requirements were not just found in one COCA standard, but surfaced through their elements, including leadership expectations, clinical training experiences, and research opportunities. Its ubiquity in the elements was noted by the AI systems. OMT remained a main distinguishing entity heavily mentioned in the COCA standards; however, most osteopathic physicians did not incorporate OMT into their regular patient care in the 21st century, as evidenced by the AOA survey results of 1,683 osteopathic physician respondents [19]. 

Only one other theme, DEI offices and approaches, surfaced as a common difference. Gemini specifically noted that COCA standards state DEI expectations are “to the extent permitted by law,” a statement notably absent from LCME standards. The COCA standard allows for a flexible approach to this area, which is a crucial characteristic, as laws and the political climate can change with each election cycle. Although a common difference cited by the systems, DEI requirements speak to other unique outcomes of utilizing this methodology. Each chatbot cited some different or overlapping COCA and LCME standards to support its conclusions. In other words, there was no uniformity in the referenced standards, indicating that the AI systems utilized some level of nuance - drawing similar conclusions, but using variable evidence (i.e., standards and elements). However, in April and in May 2025, both COCA and LCME indicated they would no longer monitor DEI efforts at medical schools. Therefore, this common difference was now moot.

Some have argued that the elements are sufficiently similar enough to question the need for two accreditation systems [13]. However, the results from these analyses showed divergences that had not been known to be explicitly cited in the literature (i.e., affiliation agreement requirements, public reporting requirements, faculty pay equity reviews, student facilities expectations, complaint requirements, time to completion and curriculum length requirements, and narrative assessment expectations, etc.). It is not to argue that these differences are additional major roadblocks to such an endeavor, just that, in fact, many differences still exist, and any merger would need to remedy such incongruencies. The AI systems were not in agreement with the significant differences chosen, showing a multitude of variances, although the significance of each finding can be debated.

As such, some responses may be deemed dubious by medical educators. We offer three examples. First, there is not a COCA- specific element that delineates when colleges must report major changes, despite an LCME element that does. Nonetheless, as mentioned, some expectations are embedded in the COCA elements, and directly on their website, signifying that any difference is likely inconsequential.

The COCA and LCME standards both require an effective system for access to confidential mental health services for students. COCA further requires 24-hour access. Despite this specific expectation, both still require evidence of an effective system, which would likely require some level of scrutiny by the accreditors that includes a review of student satisfaction. Critically, we ask, does this really rise to the level of significant difference? 

As noted, Grok 3 clarified that expectations for students to work with residents was distinct among the standards, specifically due to location of the element (LCME 3, Academic and Learning Environments versus COCA 3 Curriculum Requirements) and wording (LCME 3.1, “participates in one or more required clinical experience… with resident physicians” versus COCA 6.10, “At least one rotation… in which the student works with resident physicians”) [24,25]. Despite some potential difference in organization and intent, each element essentially distills down to a medical student requirement for exposure to GME. Again, does this distinction merit a designation as significantly different?

Although there may be some questions regarding if identified differences are significant, we argue, many identified here are still unique and distinct. We offer a few examples. As recounted, the prevalence of OMM/OMT in the COCA requirements are a clear distinction, as is the frequency of DEI expectations. COCA 6.9 specifically required clinical rotations; this also rises to a level not found in the LCME element. Likewise, some leadership positions must include individuals trained as a DO physician, an expectation obviously not found in LCME standards.

There is also at least one cross-theme finding we observed as a difference that includes expectations of public accountability in the COCA elements. Various COCA standards require student outcomes (e.g., COMLEX-USA, GME placement, student data) be made available upon request or published, presumably on the college’s website. LCME does not have practically similar expectations, with some exception given to expectations surrounding the publishing of accurate admissions requirements and the school’s accreditation status (Element 5.10 & Standard 10). 

Several limitations should be noted. The generative AI options are vast, and selecting different systems at different points in time may produce variable results. Additionally, the systems were only able to review the documents at “face value.” Their interpretation does not necessarily match the conventional thinking related to standards among site visitors or accreditors. An example would include LCME schools not using United States Medical Licensing Examination (USMLE) Step exams as an outcome metric, while COCA standards require COMLEX-USA Levels. Despite LCME not having an explicit expectation for Step 1 and 2, schools are charged to demonstrate evidence of national norms of accomplishment as detailed in LCME 8.4. Failing to require students pass these high-stakes examinations could make the programs vulnerable to noncompliance with accreditation standards. 

## Conclusions

A future analysis could include a review of the actual accreditation submission requirements and narrative questions to give a fuller picture of what may be required of each standard/element. Narrative questions accentuate potential intent of the standards and elements. Additionally, our analysis fails to capture how student data and satisfaction plays into each accreditation process. COCA, for example, requires a student survey, which is directly embedded in 11.5. LCME also requires a student survey, the independent student analysis (ISA), which is not explicitly found in LCME elements but is frequently referenced in the data collection instrument.

Finally, during this process, each noted difference presented by the chatbots needed to be considered for accuracy and compared against the standards referenced. The chatbots were relatively accurate in their comparison of the standards, despite two recognized errors, which shows overall reliability, but also some limitations to the systems. Additionally, the AI systems interpreted what “significant differences” may mean in the context of these standards. Despite these limitations, the programs returned valuable results that break down distinct differences while citing specific standards and elements. In sum, COCA and LCME present robust accreditation expectations, which are distinctly unique, not only regarding the major reference to OMT or lack thereof, but also regarding differences in several other areas as identified by the three generative AI chatbots.

## Appendices

**Table 3:** ChatGPT-4o dialogue This table represents the dialogue between the researcher and ChatGPT-4o. The left column identifies who the dialogue belongs to, while the right column shows recorded dialogue.

ChatGPT-4o Dialogue	
Researcher said:	List the ten most significant differences between the LCME and COCA standards and elements, with 1 being the most significant and 10 being the least significant. Specifically reference the standards and elements where possible.
ChatGPT said:	Top 10 Most Significant Differences Between LCME and COCA Standards 1. Osteopathic Principles and Manipulative Medicine (COCA Standard 6, Element 6.6) COCA requires all DO programs to include instruction and hands-on experience in Osteopathic Manipulative Medicine (OMM) and Osteopathic Principles and Practice (OPP) as defining elements of osteopathic education. LCME has no equivalent requirement, as it accredits allopathic (MD) programs that do not include osteopathic principles. 2. Affiliation Agreements for Clinical Training (LCME Standard 1, Element 1.4) LCME mandates formal, written agreements with all clinical sites that define responsibilities related to student education, supervision, and resources. COCA does not have this level of specificity, although it requires documentation of clinical training partnerships (Standard 6, Element 6.10). 3. Narrative Assessment in Student Evaluation (LCME Standard 9, Element 9.5) LCME requires that narrative descriptions of students' non-cognitive performance be included in assessments for all required courses and clerkships. COCA does not explicitly require narrative assessment but mandates assessments that ensure competency attainment (Standard 11, Element 11.1). 4. Financial Audit Requirement (COCA Standard 3, Element 3.4) COCA requires an annual independent financial audit to confirm financial viability. LCME does not mandate an independent audit but expects medical schools to have adequate financial resources (Standard 5, Element 5.1). 5. Structural Competence, Health Disparities, and Bias Training (LCME Standard 7, Element 7.6) LCME requires explicit training in recognizing and addressing biases, health disparities, and structural competence in the healthcare system. COCA only broadly incorporates DEI topics within its curriculum (Standard 6, Element 6.12) but does not emphasize structural competence in the same way. 6. Graduate Medical Education (GME) Placement Transparency (COCA Standard 11, Element 11.4b) COCA requires schools to publicly report GME placement rates, broken down by demographics. LCME does not mandate this level of transparency but does require schools to track graduates' outcomes. 7. Faculty Development and Pay Equity Reviews (COCA Standard 7, Element 7.8) COCA mandates a pay and rank parity review every three years to ensure fairness in faculty compensation and advancement. LCME does not have an explicit requirement for periodic faculty salary or rank equity reviews, though it requires faculty development opportunities (Standard 4, Element 4.5). 8. Mandatory Reporting of Substantial Changes (LCME Standard 5, Element 5.12) LCME requires schools to notify the accrediting body about curriculum changes, financial reductions, faculty shortages, or clinical site changes. COCA does not have a comparable element requiring notification of substantial changes before implementation. 9. Office of Diversity, Equity, and Inclusion (DEI) (COCA Standard 5, Element 5.5) COCA mandates that each school has an Office of DEI to promote recruitment and retention efforts. LCME incorporates DEI principles into various standards (Standard 3, Element 3.3) but does not require a dedicated office. 10. Study Space, Lounge Areas, and Call Rooms for Students (LCME Standard 5, Element 5.11) LCME explicitly requires medical schools to provide adequate study spaces, student lounges, secure lockers, and call rooms at all sites. COCA does not include a specific requirement for these student facilities, though it mandates adequate learning resources (Standard 4, Element 4.1). This ranking reflects the most significant differences in how each accrediting body structures its requirements, with osteopathic principles and affiliation agreements being the most impactful differences. Let me know if you need further elaboration!
Researcher said:	COCA standard 6.9 states "a com must: provide executed affiliation agreements that supports the clinical educational experience for its students." Does this conflict with significant difference number 2?
ChatGPT said:	You're correct to point that out! COCA Standard 6, Element 6.9 does require executed affiliation agreements to support clinical education, which aligns with LCME Standard 1, Element 1.4 in requiring formal agreements for clinical training sites. Revising Significant Difference #2 Instead of stating that COCA lacks specificity on affiliation agreements, the key difference should focus on the level of detail required in these agreements: LCME Standard 1, Element 1.4 outlines specific requirements for affiliation agreements, including: Medical student and faculty access to resources Primacy of the medical education program over academic affairs Appointment and assignment of faculty Student exposure policies for infectious/environmental hazards Shared responsibility for maintaining a proper learning environment COCA Standard 6, Element 6.9 mandates affiliation agreements but does not specify the same detailed elements that LCME requires. Updated Significant Difference #2 Affiliation Agreements: Level of Detail Required (LCME Standard 1, Element 1.4 vs. COCA Standard 6, Element 6.9) Both LCME and COCA require affiliation agreements for clinical education. LCME mandates specific content (e.g., academic authority, faculty roles, student safety measures). COCA requires affiliation agreements but does not provide the same level of detail on required provisions. Would you like me to update the document accordingly?
Researcher said:	Please provide the updated list of Top 10 Most Significant Differences Between LCME and COCA Standards
ChatGPT said:	Top 10 Most Significant Differences Between LCME and COCA Standards 1. Osteopathic Principles and Manipulative Medicine (COCA Standard 6, Element 6.6) COCA requires all DO programs to include instruction and hands-on experience in Osteopathic Manipulative Medicine (OMM) and Osteopathic Principles and Practice (OPP) as defining elements of osteopathic education. LCME has no equivalent requirement, as it accredits allopathic (MD) programs that do not include osteopathic principles. 2. Affiliation Agreements: Level of Detail Required (LCME Standard 1, Element 1.4 vs. COCA Standard 6, Element 6.9) Both LCME and COCA require affiliation agreements for clinical education. LCME mandates specific content (e.g., academic authority, faculty roles, student safety measures). COCA requires affiliation agreements but does not provide the same level of detail on required provisions. 3. Narrative Assessment in Student Evaluation (LCME Standard 9, Element 9.5) LCME requires that narrative descriptions of students' non-cognitive performance be included in assessments for all required courses and clerkships. COCA does not explicitly require narrative assessment but mandates assessments that ensure competency attainment (Standard 11, Element 11.1). 4. Financial Audit Requirement (COCA Standard 3, Element 3.4) COCA requires an annual independent financial audit to confirm financial viability. LCME does not mandate an independent audit but expects medical schools to have adequate financial resources (Standard 5, Element 5.1). 5. Structural Competence, Health Disparities, and Bias Training (LCME Standard 7, Element 7.6) LCME requires explicit training in recognizing and addressing biases, health disparities, and structural competence in the healthcare system. COCA only broadly incorporates DEI topics within its curriculum (Standard 6, Element 6.12) but does not emphasize structural competence in the same way. 6. Graduate Medical Education (GME) Placement Transparency (COCA Standard 11, Element 11.4b) COCA requires schools to publicly report GME placement rates, broken down by demographics. LCME does not mandate this level of transparency but does require schools to track graduates' outcomes. 7. Faculty Development and Pay Equity Reviews (COCA Standard 7, Element 7.8) COCA mandates a pay and rank parity review every three years to ensure fairness in faculty compensation and advancement. LCME does not have an explicit requirement for periodic faculty salary or rank equity reviews, though it requires faculty development opportunities (Standard 4, Element 4.5). 8. Mandatory Reporting of Substantial Changes (LCME Standard 5, Element 5.12) LCME requires schools to notify the accrediting body about curriculum changes, financial reductions, faculty shortages, or clinical site changes. COCA does not have a comparable element requiring notification of substantial changes before implementation. 9. Office of Diversity, Equity, and Inclusion (DEI) (COCA Standard 5, Element 5.5) COCA mandates that each school has an Office of DEI to promote recruitment and retention efforts. LCME incorporates DEI principles into various standards (Standard 3, Element 3.3) but does not require a dedicated office. 10. Study Space, Lounge Areas, and Call Rooms for Students (LCME Standard 5, Element 5.11) LCME explicitly requires medical schools to provide adequate study spaces, student lounges, secure lockers, and call rooms at all sites. COCA does not include a specific requirement for these student facilities, though it mandates adequate learning resources (Standard 4, Element 4.1). This revised list now accurately reflects the distinction between LCME and COCA affiliation agreements while retaining the most impactful differences in medical education accreditation. Let me know if you need further refinements!

**Table 4:** Gemini 2.0 Flash dialogue This table represents the dialogue between the researcher and Gemini 2.0 Flash. The left column identifies who the dialogue belongs to, while the right column shows recorded dialogue.

Gemini Dialogue	
Researcher said:	List the ten most significant differences between the LCME and COCA standards and elements, with 1 being the most significant and 10 being the least significant. Specifically reference the standards and elements where possible.
Gemini said:	Based on a review of the documents, here are ten significant differences between LCME and COCA standards and elements, ranked from most to least significant: 1. Osteopathic Principles and Practice/Osteopathic Manipulative Treatment (OMM/OPP) Training: COCA mandates specific instruction in osteopathic principles and practice, including OMM, throughout the curriculum. This is a fundamental distinction, as it defines the core identity of osteopathic medical education. LCME standards do not include this, as they pertain to allopathic medical education. 2. ** требования к образованию декана**: COCA mandates that the dean of a COM have an earned DO degree, an unrestricted medical license at some point, AOA or ABMS board certification at some time, and at least five years of academic leadership experience within the last ten years. LCME, while requiring the dean to be qualified by education, training, and experience, does not specify the degree or type of licensure. 3. Primary Care Leadership: COCA requires that the leadership of the COM’s clinical education include one or more actively licensed osteopathic physicians who are AOA or ABMS board-certified in a primary care discipline, with proven experience in teaching and academic leadership in a medical education setting. LCME does not have this requirement. 4. Research and Scholarly Activity in OMM/OPP: COCA requires that a COM demonstrates how its research and/or scholarly activity includes and/or incorporates osteopathic manipulative medicine (OMM) and osteopathic principles and practice (OPP). LCME does not have a comparable requirement. 5. ** различное отношение к DEI**: While both COCA and LCME emphasize diversity, equity, and inclusion (DEI), COCA standards frequently include the phrase "to the extent permitted by law" within DEI-related elements. This qualification is not present in the LCME standards. Additionally, COCA requires a strategic plan for DEI, whereas LCME does not. 6. ** требования к жалобам**: COCA mandates that the accreditation standard complaint filing process must also include a process for filing confidential complaints with the COCA and the contact information of the COCA. LCME does not specify this. 7. ** требования к клиническому образованию**: COCA is more specific about the requirements for core clinical rotations, specifying that core rotations must be taught in the third year of the curriculum by faculty who hold (or have held) board certification, or be board eligible, in the listed specialty. LCME does not specify this. 8. Faculty Credentials at Non-Core Rotation Sites: COCA requires a mechanism to review and approve credentials of physician faculty that have never been board-certified or are not board-eligible by the AOA or ABMS at non-core rotation sites. LCME does not specify this. 9. Student Debt Load and Default Rates: COCA mandates that a COM must make available by request four years of student debt load and student loan default rates, including demographic data. While LCME addresses financial aid and debt management counseling, it does not explicitly require the publication of debt load and default rates. 10. ** требования к академической свободе**: COCA explicitly requires that a COM include in its publications policies regarding academic freedom, approved by the COM’s governing board, and that these policies apply to all campus locations. While LCME emphasizes faculty roles and responsibilities, it does not explicitly mandate published policies on academic freedom.
Researcher said:	Some of the text is not discernable and appears to be in a language other than English. Can this be corrected to English? For example: требования к образованию декана
Gemini said:	You are absolutely correct to point that out. There was an instance where the translation from the original document was not correctly rendered. Here is the corrected list, with the inaccurate text replaced with the correct English translation: Based on a review of the documents, here are ten significant differences between LCME and COCA standards and elements, ranked from most to least significant: 1. Osteopathic Principles and Practice/Osteopathic Manipulative Treatment (OMM/OPP) Training: COCA mandates specific instruction in osteopathic principles and practice, including OMM, throughout the curriculum. This is a fundamental distinction, as it defines the core identity of osteopathic medical education. LCME standards do not include this, as they pertain to allopathic medical education. 2. Dean’s Qualifications: COCA mandates that the dean of a COM have an earned DO degree, an unrestricted medical license at some point, AOA or ABMS board certification at some time, and at least five years of academic leadership experience within the last ten years. LCME, while requiring the dean to be qualified by education, training, and experience, does not specify the degree or type of licensure. 3. Primary Care Leadership: COCA requires that the leadership of the COM’s clinical education include one or more actively licensed osteopathic physicians who are AOA or ABMS board-certified in a primary care discipline, with proven experience in teaching and academic leadership in a medical education setting. LCME does not have this requirement. 4. Research and Scholarly Activity in OMM/OPP: COCA requires that a COM demonstrates how its research and/or scholarly activity includes and/or incorporates osteopathic manipulative medicine (OMM) and osteopathic principles and practice (OPP). LCME does not have a comparable requirement. 5. Different Approaches to DEI: While both COCA and LCME emphasize diversity, equity, and inclusion (DEI), COCA standards frequently include the phrase "to the extent permitted by law" within DEI-related elements. This qualification is not present in the LCME standards. Additionally, COCA requires a strategic plan for DEI, whereas LCME does not. 6. Complaint Requirements: COCA mandates that the accreditation standard complaint filing process must also include a process for filing confidential complaints with the COCA and the contact information of the COCA. LCME does not specify this. 7. Clinical Education Requirements: COCA is more specific about the requirements for core clinical rotations, specifying that core rotations must be taught in the third year of the curriculum by faculty who hold (or have held) board certification, or be board eligible, in the listed specialty. LCME does not specify this. 8. Faculty Credentials at Non-Core Rotation Sites: COCA requires a mechanism to review and approve credentials of physician faculty that have never been board-certified or are not board-eligible by the AOA or ABMS at non-core rotation sites. LCME does not specify this. 9. Student Debt Load and Default Rates: COCA mandates that a COM must make available by request four years of student debt load and student loan default rates, including demographic data. While LCME addresses financial aid and debt management counseling, it does not explicitly require the publication of debt load and default rates. 10. Academic Freedom Requirements: COCA explicitly requires that a COM include in its publications policies regarding academic freedom, approved by the COM’s governing board, and that these policies apply to all campus locations. While LCME emphasizes faculty roles and responsibilities, it does not explicitly mandate published policies on academic freedom.
Researcher said:	Please revi[s]e the list referencing the specific LCME and COCA elements.
Gemini said:	Okay, I can provide a revised list that includes references to the specific LCME and COCA standards and elements. Here are the ten most significant differences between LCME and COCA standards and elements, ranked from most to least significant, with specific references: 1. Osteopathic Principles and Practice/Osteopathic Manipulative Treatment (OMM/OPP) Training: COCA mandates specific instruction in osteopathic principles and practice, including OMM, throughout the curriculum. This is a fundamental distinction, as it defines the core identity of osteopathic medical education. LCME standards do not include this, as they pertain to allopathic medical education. COCA reference: Element 6.6, "Principles of Osteopathic Medicine (CORE)" and Element 6.10, “Clinical Experience” LCME has no comparable standard or element. 2. Dean’s Qualifications: COCA mandates that the dean of a COM have an earned DO degree, an unrestricted medical license at some point, AOA or ABMS board certification at some time, and at least five years of academic leadership experience within the last ten years. LCME, while requiring the dean to be qualified by education, training, and experience, does not specify the degree or type of licensure. COCA reference: Element 2.1, "Dean Qualifications (CORE)" LCME reference: Standard 2.2, "Dean’s Qualifications" 3. Primary Care Leadership: COCA requires that the leadership of the COM’s clinical education include one or more actively licensed osteopathic physicians who are AOA or ABMS board-certified in a primary care discipline, with proven experience in teaching and academic leadership in a medical education setting. LCME does not have this requirement. COCA reference: Element 7.4, "Primary Care Leadership" LCME has no comparable standard or element. 4. Research and Scholarly Activity in OMM/OPP: COCA requires that a COM demonstrates how its research and/or scholarly activity includes and/or incorporates osteopathic manipulative medicine (OMM) and osteopathic principles and practice (OPP). LCME does not have a comparable requirement. COCA reference: Element 8.3, "OMM/OPP Research and Scholarly Activity (CORE)" LCME has no comparable standard or element. 5. Different Approaches to DEI: While both COCA and LCME emphasize diversity, equity, and inclusion (DEI), COCA standards frequently include the phrase "to the extent permitted by law" within DEI-related elements. This qualification is not present in the LCME standards. Additionally, COCA requires a strategic plan for DEI, whereas LCME does not. COCA references: Element 1.1, “Program Mission”, Element 1.2, “Strategic Plan”, Element 4.1, “Facilities (CORE)”, Element 5.2, “Diversity”, Element 6.12, “Diversity, Equity, and Inclusion (DEI) Curriculum (CORE)”, Element 7.9, “Diversity, Equity, and Inclusion (DEI) Training”, and Standard 11, “Program and Student Assessment and Outcomes” LCME references: Standard 3, "Academic and Learning Environments", Standard 3.3, "Diversity Programs and Partnerships", and Standard 7.6, “Structural Competence, Cultural Competence, and Health Inequities” 6. Complaint Requirements: COCA mandates that the accreditation standard complaint filing process must also include a process for filing confidential complaints with the COCA and the contact information of the COCA. LCME does not specify this. COCA reference: Element 2.4, "Accreditation Standard Complaint Policies and Procedures (CORE)" LCME has no comparable standard or element. 7. Clinical Education Requirements: COCA is more specific about the requirements for core clinical rotations, specifying that core rotations must be taught in the third year of the curriculum by faculty who hold (or have held) board certification, or be board eligible, in the listed specialty. LCME does not specify this. COCA reference: Element 6.9, "Clinical Education (CORE)" LCME reference: Standard 6.2, "Required Clinical Experiences" 8. Faculty Credentials at Non-Core Rotation Sites: COCA requires a mechanism to review and approve credentials of physician faculty that have never been board-certified or are not board-eligible by the AOA or ABMS at non-core rotation sites. LCME does not specify this. COCA reference: Element 7.1, "Faculty and Staff Resources and Qualifications (CORE)" LCME has no comparable standard or element. 9. Student Debt Load and Default Rates: COCA mandates that a COM must make available by request four years of student debt load and student loan default rates, including demographic data. While LCME addresses financial aid and debt management counseling, it does not explicitly require the publication of debt load and default rates. COCA reference: Element 11.3, "Student Debt Outcomes" LCME reference: Standard 12.1, "Financial Aid/Debt Management Counseling/Student Educational Debt" 10. Academic Freedom Requirements: COCA explicitly requires that a COM include in its publications policies regarding academic freedom, approved by the COM’s governing board, and that these policies apply to all campus locations. While LCME emphasizes faculty roles and responsibilities, it does not explicitly mandate published policies on academic freedom. COCA reference: Element 12.8, "Academic Freedom" LCME has no comparable standard or element.

**Table 5:** Dialogue with Grok 3 This table represents the dialogue between the researcher and Grok 3. The left column identifies who the dialogue belongs to, while the right column shows recorded dialogue.

Grok Dialogue	
Researcher said:	List the ten most significant differences between the LCME and COCA standards and elements, with 1 being the most significant and 10 being the least significant. Specifically reference the standards and elements where possible.
Grok said:	Below is a list of the ten most significant differences between the LCME (Liaison Committee on Medical Education) standards for allopathic medical schools and the COCA (Commission on Osteopathic College Accreditation) standards for osteopathic medical schools, ranked from 1 (most significant) to 10 (least significant). The ranking is based on the impact on educational philosophy, curriculum, institutional structure, and student outcomes, with specific references to standards and elements where applicable. Ten Most Significant Differences: 1. Osteopathic Core Competencies and OMM/OPP Emphasis (COCA 6.4, 6.6, 7.5 vs. LCME Absence) Significance: This is the most fundamental difference, reflecting the distinct identity of osteopathic medicine. COCA mandates teaching and assessing seven osteopathic core competencies, including osteopathic principles and practice (OPP) and osteopathic manipulative treatment (OMT) (6.4), with required instruction in OPP/OMM each year (6.6) led by a specialized DO with board certification (7.5). LCME has no equivalent focus on osteopathic philosophy or manual techniques, emphasizing general medical competencies instead. Impact: Defines the philosophical and practical divide between DO and MD education. 2. COMLEX-USA Examination Requirement (COCA 11.4a vs. LCME Absence) Significance: COCA requires students to pass COMLEX-USA Levels 1 and 2 before graduation, with public reporting of pass rates (11.4a), tying licensure preparation to osteopathic-specific assessment. LCME does not mandate a specific exam (e.g., USMLE) for graduation, leaving it to institutional discretion. Impact: Directly affects student assessment, graduation requirements, and public accountability. 3. Diversity, Equity, and Inclusion (DEI) Office and Training (COCA 5.5, 7.9 vs. LCME 3.3) Significance: COCA mandates a dedicated Office of DEI with a strategic plan (5.5) and annual DEI training for faculty and staff (7.9), embedding DEI structurally. LCME addresses DEI through recruitment and retention policies (3.3) but does not require a specific office or mandatory training. Impact: Reflects a stronger institutional commitment to DEI in COCA, influencing culture and operations. 4. OMM/OPP Research Requirement (COCA 8.3 vs. LCME 3.2, 7.3) Significance: COCA requires research and scholarly activity to include OMM and OPP (8.3), aligning with osteopathic identity. LCME encourages research opportunities (3.2) and scientific method instruction (7.3) but lacks specificity about content focus. Impact: Shapes the research mission and output of osteopathic schools distinctly from allopathic ones. 5. Minimum Program Duration (LCME 6.8 vs. COCA 6.3) Significance: LCME specifies a minimum of 130 weeks of instruction (6.8), ensuring a standardized duration. COCA limits completion to 150% of standard time (6 years) (6.3) but does not define a minimum instructional period, allowing flexibility. Impact: Affects curriculum structure and consistency across institutions. 6. Resident Participation in Student Education (LCME 3.1 vs. COCA 6.10) Significance: LCME mandates clinical experiences with resident physicians in accredited GME programs (3.1), emphasizing interprofessional training. COCA requires one rotation with residents (6.10) but does not mandate it as a core component across all experiences. Impact: Influences the integration of GME into undergraduate medical education. 7. Primary Care and OMM/OPP Leadership (COCA 7.4, 7.5 vs. LCME Absence) Significance: COCA requires clinical education leadership to include board-certified primary care osteopathic physicians (7.4) and a full-time OMM/OPP leader with specific credentials (7.5). LCME does not specify leadership roles by specialty or require osteopathic expertise. Impact: Reinforces osteopathic focus on primary care and manual medicine through leadership. 8. Narrative Assessment Requirement (LCME 9.5 vs. COCA Absence) Significance: LCME requires narrative descriptions of student performance in required courses/clerkships when interaction permits (9.5), enhancing qualitative feedback. COCA mentions formative/summative assessment (7.7) but does not mandate narrative components. Impact: Affects the depth and type of student evaluation feedback. 9. GME Support and Osteopathic Recognition (COCA 10.2, 10.3 vs. LCME Absence) Significance: COCA requires mechanisms to assist GME programs in achieving accreditation (10.2) and osteopathic recognition (10.3), extending responsibility beyond undergraduate education. LCME focuses on undergraduate education without explicit GME support requirements. Impact: Expands COCA’s scope to influence postgraduate training. 10. Mental Health Services Availability (COCA 9.8 vs. LCME 12.3) Significance: COCA mandates 24/7/365 confidential mental health access (9.8), ensuring constant support. LCME requires effective counseling (12.3) but does not specify round-the-clock availability. Impact: Enhances student well-being support in COCA, though the practical difference may vary by implementation. Rationale for Ranking 1. Rank 1-4: These differences (osteopathic focus, COMLEX, DEI structure, OMM research) are foundational, shaping the core identity, assessment, and institutional priorities of osteopathic vs. allopathic education. 2. Rank 5-7: Program duration, resident interaction, and leadership roles impact curriculum design and faculty structure but are less defining of the overall educational philosophy. 3. Rank 8-10: Narrative assessment, GME support, and mental health availability influence student experience and outcomes but are operational rather than philosophical distinctions. These differences highlight the osteopathic emphasis on manual medicine and holistic care in COCA, contrasted with LCME’s broader, allopathic framework focused on standardized assessment and flexibility.
Researcher said:	In number 8, the response cites COCA element 7.7 as mentioning formative and summative feedback. Is that the correct element or should the reference instead be to standard 7?
Grok said:	You’re correct to question the reference. I apologize for the oversight in my previous response. The citation to **COCA Element 7.7** as mentioning formative and summative feedback is incorrect. Element 7.7 actually pertains to the "Faculty Association" and focuses on providing a representative forum for faculty participation, not student assessment. The correct reference should indeed be to **COCA Standard 7** as a whole, which is titled "Faculty and Staff." Specifically, the relevant element within Standard 7 that addresses formative and summative assessment is **Element 7.1: Faculty and Staff Resources and Qualifications (CORE)**. However, a more precise connection to assessment lies in the introductory text of **Standard 7**, which states: > "A COM must ensure that its medical education program includes a comprehensive, fair, and uniform system of formative and summative medical student assessment and protects medical students’ and patients’ safety by ensuring that all persons who teach, supervise, and/or assess medical students are adequately prepared for those responsibilities." This introductory paragraph of Standard 7 sets the context for assessment but does not provide specific details about formative or summative feedback timing or methods, unlike LCME’s **Element 9.5 (Narrative Assessment)**, which explicitly mandates narrative descriptions of student performance. To clarify and correct the record: - **Incorrect Reference**: COCA Element 7.7 (Faculty Association) does not mention formative or summative feedback. - **Correct Reference**: COCA Standard 7 (introductory text) broadly mentions a "comprehensive, fair, and uniform system of formative and summative medical student assessment," but no specific element under Standard 7 details narrative or timing requirements as LCME 9.5 does. Thus, in the context of difference #8 ("Narrative Assessment Requirement"), the comparison should reference **COCA Standard 7** (introductory text) rather than Element 7.7. Here’s the revised statement for accuracy: --- ### Revised Difference #8 **Narrative Assessment Requirement (LCME 9.5 vs. COCA Standard 7)** -**Significance**: LCME requires narrative descriptions of student performance in required courses/clerkships when interaction permits (9.5), enhancing qualitative feedback. COCA’s Standard 7 mentions a "comprehensive, fair, and uniform system of formative and summative medical student assessment" in its introductory text but does not mandate narrative components or specify assessment details in any element. **Impact**: Affects the depth and type of student evaluation feedback. ---Thank you for catching this. This correction ensures the comparison accurately reflects the content of the COCA standards.
Researcher said:	please provide more context for number 6 and how lcme 3.1 and coca 6.10 are different in nature.
Grok said:	Below, I’ll provide more context for Difference #6: Resident Participation in Student Education (LCME 3.1 vs. COCA 6.10) from the list of the ten most significant differences between the LCME and COCA standards. I’ll explain the specific requirements of each standard/element, highlight their differences in nature, and elaborate on their implications for medical education. Difference #6: Resident Participation in Student Education (LCME 3.1 vs. COCA 6.10) LCME Element 3.1: Resident Participation in Medical Student Education Text: "Each medical student in a medical education program participates in one or more required clinical experiences conducted in a health care setting in which he or she works with resident physicians currently enrolled in an accredited program of graduate medical education." Context: LCME 3.1 is part of Standard 3: Academic and Learning Environments, which focuses on ensuring professional, respectful, and stimulating environments for medical education. This element mandates that *all medical students* engage in at least one *required clinical experience* (e.g., a core clerkship like internal medicine or surgery) where they work directly with resident physicians who are part of an accredited graduate medical education (GME) program (e.g., ACGME-accredited residencies). The emphasis is on integrating undergraduate medical education (UME) with GME, exposing students to the hierarchical team-based structure of clinical practice, and fostering interprofessional learning early in their training. This requirement ensures consistent exposure to residents as teachers and role models across the curriculum, typically in hospital-based settings where residents are prevalent. Key Features: Applies to *all students* in *required* clinical experiences (not optional or elective). Specifies residents must be in *accredited GME programs*, ensuring a standardized level of training and oversight. Broadly integrated into the curriculum, implying multiple opportunities rather than a single instance. COCA Element 6.10: Clinical Experience Text: "A COM and any branch campus must ensure that each student’s rotations prior to the fourth-year clinical clerkships include the following experiences: 1) at least one rotation conducted in a health care setting in which the student works with resident physicians currently enrolled in an accredited program of graduate medical education; 2) at least one rotation under the supervision of an osteopathic physician; and 3) more than one rotation in an inpatient setting." Context: COCA 6.10 falls under Standard 6: Curriculum, which outlines the design and implementation of the osteopathic medical curriculum to achieve educational objectives, including osteopathic core competencies. This element specifies three distinct clinical experience requirements that must occur *prior to fourth-year clerkships* (i.e., typically during the third year, when core and required rotations like family medicine or internal medicine are completed). The requirement to work with resident physicians in an accredited GME program is just one of three components, alongside an osteopathic physician-led rotation and multiple inpatient experiences. This reflects COCA’s broader goal of ensuring diverse clinical exposure while maintaining an osteopathic identity (e.g., the DO supervision requirement). Key Features: Mandates *at least one rotation* with residents, but this is not required across all clinical experiences. Limited to rotations *before the fourth year*, focusing on early clinical training. Includes additional requirements (DO supervision, inpatient settings), diluting the focus on resident interaction compared to LCME. How LCME 3.1 and COCA 6.10 Differ in Nature 1. Scope and Breadth: LCME 3.1: Requires resident participation in *one or more required clinical experiences* without limiting it to a specific year or rotation type. This implies a pervasive integration of resident interaction throughout the clinical curriculum (e.g., across multiple clerkships like surgery, pediatrics, etc.), reflecting the allopathic emphasis on hospital-based, residency-driven training environments. COCA 6.10: Specifies *at least one rotation* with residents, restricted to the pre-fourth-year phase (typically third-year core/required rotations). It’s a narrower requirement, ensuring a minimum exposure rather than a consistent presence across the curriculum. This reflects osteopathic medicine’s varied training settings, which may include community-based or outpatient sites where residents are less common. 2. Focus and Priority: LCME 3.1: Places a singular, explicit emphasis on resident involvement as a critical component of clinical education, aligning with the allopathic model where residents are key educators in teaching hospitals. It prioritizes interprofessional learning with GME trainees as a core educational strategy. COCA 6.10: Treats resident interaction as one of three co-equal requirements, alongside osteopathic physician supervision and inpatient exposure. The focus is split, with resident interaction competing with osteopathic identity (DO supervision) and setting diversity (inpatient focus), suggesting it’s less central to the osteopathic educational philosophy. 3. Integration into Curriculum: LCME 3.1: Implies a systemic integration, as "required clinical experiences" encompass all core clerkships (e.g., LCME 6.2 defines required patient encounters and skills). This ensures all students repeatedly encounter residents, enhancing continuity and depth of exposure. COCA 6.10: Limits the requirement to a single instance before the fourth year, potentially fulfilled by one rotation (e.g., internal medicine). Subsequent rotations (including fourth-year electives) are not obligated to involve residents, reducing the overall emphasis on GME integration. 4. Educational Philosophy: LCME 3.1: Reflects an allopathic model heavily reliant on academic medical centers and teaching hospitals, where residents are integral to the clinical team and student education. It assumes a standardized, hospital-centric training environment. COCA 6.10: Accommodates the osteopathic model’s broader training landscape, which includes community hospitals, rural sites, and outpatient settings where residents may not always be present. The additional DO supervision requirement underscores osteopathic distinctiveness over GME alignment. 5. Flexibility vs. Specificity: LCME 3.1: Offers flexibility in implementation (one or more experiences) but sets a firm expectation of resident involvement across required settings, ensuring consistency. COCA 6.10: Provides specific, checklist-style criteria (one rotation with residents, one with a DO, multiple inpatient) but allows flexibility in how much resident exposure occurs beyond the minimum, potentially leading to variability across COMs. Implications of the Difference 1. Educational Experience: LCME students are guaranteed broader and more consistent exposure to residents, fostering mentorship, team-based learning, and preparation for residency in academic settings. COCA students may have more variable exposure, with a single resident-involved rotation meeting the requirement, potentially limiting their interprofessional experience with GME trainees. 2. Institutional Burden: LCME’s requirement demands robust affiliations with teaching hospitals hosting accredited residencies, which may challenge smaller or rural schools. COCA’s narrower scope eases this burden, aligning with osteopathic schools’ diverse clinical partnerships, but may reduce alignment with GME culture. 3. Osteopathic Identity: COCA’s inclusion of DO supervision alongside resident interaction reinforces osteopathic principles, while LCME’s focus solely on residents aligns with a generalist, allopathic framework. Conclusion The difference in nature between LCME 3.1 and COCA 6.10 lies in their scope, priority, and philosophical underpinnings. LCME 3.1 embeds resident participation as a pervasive, high-priority feature of clinical education, reflecting allopathic reliance on GME integration. COCA 6.10 treats it as a minimum requirement within a broader set of clinical exposure goals, balancing osteopathic identity and flexibility. This ranks as #6 in significance because it shapes clinical training environments and interprofessional exposure, though it’s less defining than osteopathic-specific elements (e.g., OMM) or assessment mandates (e.g., COMLEX).
Researcher said:	Please provide an updated list
Grok said:	Below is an updated list of the ten most significant differences between the LCME (Liaison Committee on Medical Education) standards for allopathic medical schools and the COCA (Commission on Osteopathic College Accreditation) standards for osteopathic medical schools. This revision corrects the reference in Difference #8 from COCA Element 7.7 to COCA Standard 7 (introductory text) based on the earlier clarification and incorporates additional context for Difference #6 (LCME 3.1 vs. COCA 6.10) as requested. The ranking from 1 (most significant) to 10 (least significant) remains consistent with the original reasoning, focusing on impact on educational philosophy, curriculum, institutional structure, and student outcomes. Updated Ten Most Significant Differences 1. Osteopathic Core Competencies and OMM/OPP Emphasis (COCA 6.4, 6.6, 7.5 vs. LCME Absence) Description: COCA mandates teaching and assessing seven osteopathic core competencies, including osteopathic principles and practice (OPP) and osteopathic manipulative treatment (OMT) (6.4), with required instruction in OPP/OMM each year (6.6) led by a specialized DO with board certification (7.5). LCME has no equivalent focus on osteopathic philosophy or manual techniques, emphasizing general medical competencies. Significance: Defines the philosophical and practical divide between DO and MD education, making it the most foundational difference. 2. COMLEX-USA Examination Requirement (COCA 11.4a vs. LCME Absence) Description: COCA requires students to pass COMLEX-USA Levels 1 and 2 before graduation, with public reporting of pass rates (11.4a). LCME does not mandate a specific exam (e.g., USMLE) for graduation, leaving it to institutional discretion. Significance: Directly affects student assessment, graduation requirements, and public accountability, tying osteopathic education to a specific licensure pathway. 3. Diversity, Equity, and Inclusion (DEI) Office and Training (COCA 5.5, 7.9 vs. LCME 3.3) Description: COCA mandates a dedicated Office of DEI with a strategic plan (5.5) and annual DEI training for faculty and staff (7.9). LCME addresses DEI through recruitment and retention policies (3.3) but does not require a specific office or mandatory training. Significance: Reflects a stronger structural commitment to DEI in COCA, influencing institutional culture and operations. 4. OMM/OPP Research Requirement (COCA 8.3 vs. LCME 3.2, 7.3) Description: COCA requires research and scholarly activity to include OMM and OPP (8.3). LCME encourages research opportunities (3.2) and scientific method instruction (7.3) but lacks specificity about content focus. Significance: Shapes the research mission and output of osteopathic schools distinctly, reinforcing their unique identity. 5. Minimum Program Duration (LCME 6.8 vs. COCA 6.3) Description: LCME specifies a minimum of 130 weeks of instruction (6.8). COCA limits completion to 150% of standard time (6 years) (6.3) but does not define a minimum instructional period. Significance: Affects curriculum structure and consistency, with LCME ensuring a standardized duration. 6. Resident Participation in Student Education (LCME 3.1 vs. COCA 6.10) Description: LCME mandates that all medical students participate in one or more required clinical experiences with resident physicians in accredited GME programs (3.1), implying pervasive integration throughout the clinical curriculum. COCA requires at least one rotation with residents before the fourth year, alongside a rotation with an osteopathic physician and multiple inpatient experiences (6.10), treating it as a minimum requirement within broader exposure goals. Difference in Nature: LCME emphasizes consistent resident interaction across required clerkships, reflecting allopathic reliance on teaching hospitals and GME integration. COCA limits it to a single pre-fourth-year instance, balancing it with osteopathic identity (DO supervision) and setting diversity, accommodating varied training environments. Significance: Influences interprofessional exposure and clinical training alignment with GME, though less defining than osteopathic-specific elements. 7. Primary Care and OMM/OPP Leadership (COCA 7.4, 7.5 vs. LCME Absence) Description: COCA requires clinical education leadership to include board-certified primary care osteopathic physicians (7.4) and a full-time OMM/OPP leader with specific credentials (7.5). LCME does not specify leadership roles by specialty or require osteopathic expertise. Significance: Reinforces osteopathic focus on primary care and manual medicine through leadership structure. 8. Narrative Assessment Requirement (LCME 9.5 vs. COCA Standard 7) Description: LCME requires narrative descriptions of student performance in required courses/clerkships when interaction permits (9.5). COCA’s Standard 7 (introductory text) mentions a "comprehensive, fair, and uniform system of formative and summative medical student assessment" but does not mandate narrative components or specify details. Significance: Affects the depth and type of student evaluation feedback, with LCME providing a richer qualitative assessment tool. 9. GME Support and Osteopathic Recognition (COCA 10.2, 10.3 vs. LCME Absence) Description: COCA requires mechanisms to assist GME programs in achieving accreditation (10.2) and osteopathic recognition (10.3). LCME focuses on undergraduate education without explicit GME support requirements. Significance: Extends COCA’s scope to influence postgraduate training, reflecting a continuum of osteopathic education. 10. Mental Health Services Availability (COCA 9.8 vs. LCME 12.3) Description: COCA mandates 24/7/365 confidential mental health access (9.8). LCME requires effective counseling (12.3) but does not specify round-the-clock availability. Significance: Enhances student well-being support in COCA, though practical differences depend on implementation. Notes on Updates Difference #8: Corrected the reference from COCA Element 7.7 (Faculty Association) to COCA Standard 7 (introductory text), reflecting the accurate location of the formative/summative assessment mention. This does not alter its ranking, as the significance stems from LCME’s specific narrative requirement vs. COCA’s general assessment framework. Difference #6: Expanded context clarifies the broader scope and priority of LCME 3.1 (pervasive resident integration) vs. the narrower, multi-faceted requirement of COCA 6.10 (minimum resident exposure plus osteopathic and inpatient focus). Its #6 ranking remains appropriate, as it impacts clinical training but is less foundational than osteopathic philosophy or assessment mandates. This updated list ensures accuracy and provides deeper insight into the resident participation difference, maintaining the original framework’s intent.
